# A robust acid-resistant chelating polymer for enhanced stabilization of lead ions in fly ash

**DOI:** 10.1186/s13065-024-01209-z

**Published:** 2024-05-23

**Authors:** Qi Wang, Huiyu Yan, Linyan Yao, Ying Guo, Jianxi Xiao

**Affiliations:** State Key Laboratory of Applied Organic Chemistry, College of Chemistry and Chemical Engineering, Lanzhou University, Lanzhou, 730000 Gansu People’s Republic of China

**Keywords:** Fly ash, Pb^2+^, Polymer, Chelator, Municipal solid waste incineration, Environmental management

## Abstract

Fly ash derived from municipal solid waste incinerators (MSWIs) harbors significant quantities of heavy metals with high leaching toxicity, resulting in detrimental environmental effects. Pb^2+^ in fly ash is the ion most likely to exceed permissible levels. However, chemical stabilization methods demonstrate poor efficacy in stabilizing Pb^2+^ under acidic conditions. Herein, we have developed a robust acid-resistant chelating polymer (25DTF) for enhanced stabilization of Pb^2+^ in fly ash. 25DTF was synthesized through the reaction of formaldehyde with 2,5-dithiourea. 25DTF exhibited remarkable chelation efficiency, nearing 100%, for Pb^2+^ in fly ash. 25DTF demonstrated exceptional chelation efficiency, surpassing 99.9%, when interacting with Pb^2+^ in fly ash at pH ≤ 7. Even under acidic conditions, 25DTF effectively prevented the secondary dissolution of Pb^2+^. Additionally, it indicated outstanding Pb^2+^ chelation efficiency across diverse regions of China. The 25DTF chelating agent shows considerable potential in alleviating metal ion contamination in soil, wastewater, and urban environmental management, thereby fostering advancements in environmental stewardship.

**Supplementary Information:**

The online version contains supplementary material available at 10.1186/s13065-024-01209-z.

## Introduction

Municipal solid waste (MSW) has become a significant threat to environmental stability and sustainable progress, attributable to the expansion of economies and urban populations [1]. The current global waste production surpasses 2 billion tons annually and is projected to escalate to 3.4 billion tons by the year 2050 [2, 3]. Municipal solid waste incinerator (MSWI) technology is widely recognized and highly valued as a waste management solution, owing to its superior treatment capacity, excellent waste reduction capabilities, integrated environmental safety measures, and heat recovery benefits. [4, 5]. However, MSWI generates fly ash containing heavy metals with high leaching toxicity, including Cu^2+^, Zn^2+^, and Pb^2+^, thereby raising environmental concerns [6–8].

Heavy metals exhibit a propensity for continuous migration and environmental alteration. Contaminated soil and water environments lack inherent self-purification mechanisms, while microorganisms are unable to degrade heavy metals, allowing them to accumulate within the biological chain. Mishandling of fly ash can result in the dispersion of pollutants and heavy metals into the environment, posing significant risks to both human health and the integrity of the natural ecosystem. Pb^2+^ is widely acknowledged as a toxic pollutant on a global scale and is frequently found in fly ash. According to the GB16889-2008 “Pollution Control Standards for Domestic Waste Landfills”, the allowable Pb^2+^ content in fly ash leachate should not exceed 0.25 mg/L [9]. Hence, it is imperative to develop methods for treating heavy metal ions in fly ash.

Various methods have been developed to address Pb^2+^ in fly ash, including solidification using cement, melt solidification, and chemical stabilization techniques. The method of cement solidification accomplishes Pb^2+^ removal by forming stable compounds on the surface of cement-hydrated silicate colloids [10–13]. However, this method exhibits weak integration capacity and poor stability. Melt solidification is another feasible alternative [14], but it is related to elevated energy expenditure and cost [15–17]. Therefore, it is important to develop a cost-effective and efficacious approach for treating Pb^2+^ present in fly ash.

Chemical stability has attracted much attention due to its advantages such as harmlessness of waste and small volume increase after treatment [18–20]. Na_2_S was utilized to remove heavy metal ions from soil, achieving a removal efficiency for Cd^2+^ ranging from 69 to 84% [21, 22]. Chelating agents such as strong inorganic acids, ammonia salts and organic acids have the advantages of low cost and simplicity, eliminating hazardous metals from waste fly ash [23, 24]. However, conventional stabilization technologies still have some shortcomings that require resolution, including the challenge of stably chelating Pb^2+^ under acidic conditions, achieving long-term stability of heavy metals across a wide pH range, and the necessity for large quantities of reagents. The leaching behavior of heavy metals is influenced by factors such as ash properties, leachate pH, leachate type, and microorganisms. The issue of leaching heavy metal ions from fly ash continues to be a focal point of current research. The quest for efficient chelating agents to immobilize Pb^2+^ in fly ash while mitigating secondary leaching under acidic conditions remains a significant challenge.

**Scheme 1:**
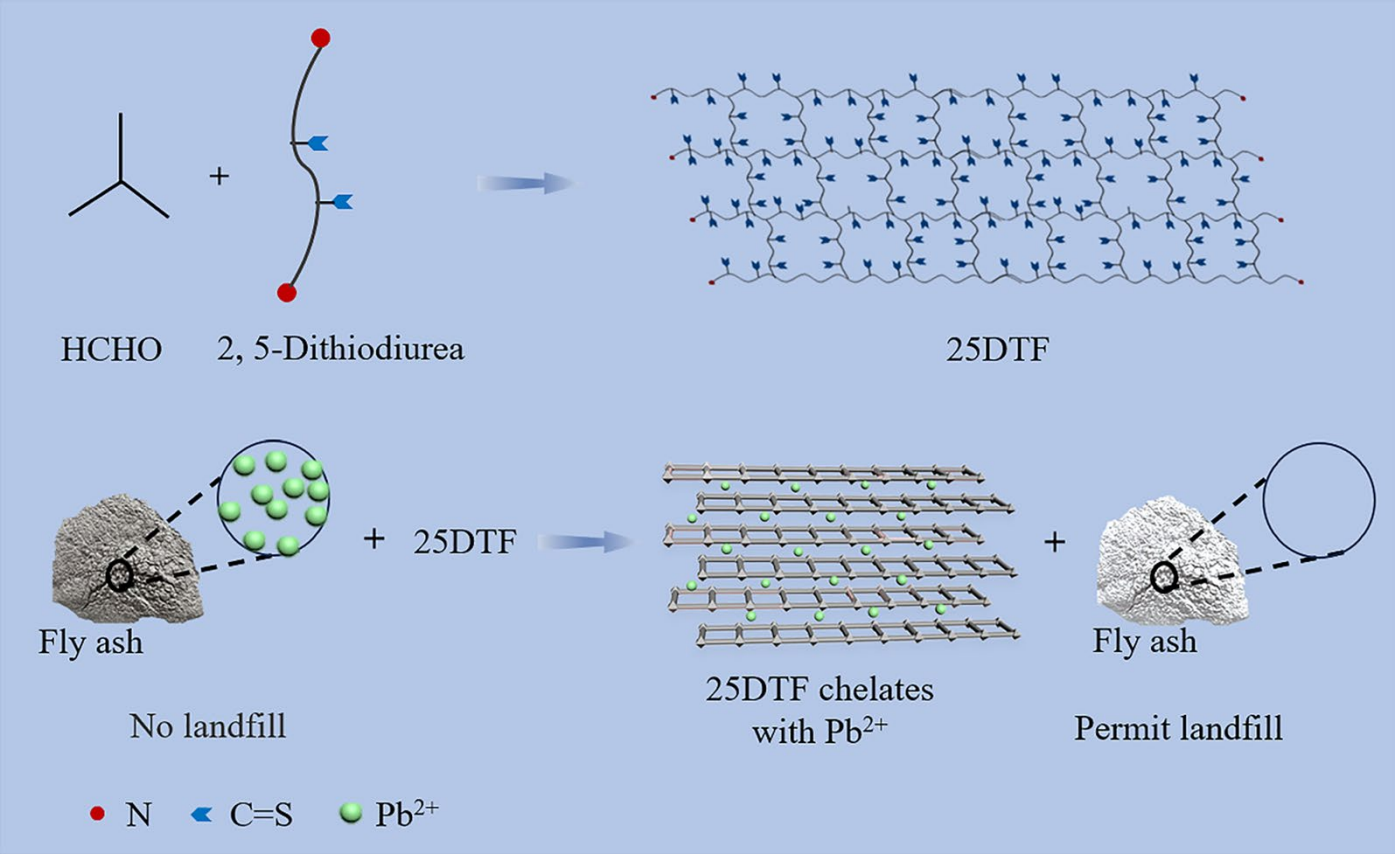
Schematic diagram of the construction of a novel polymer chelating agent for the treatment of Pb^2+^ in fly ash by the reaction between formaldehyde and 2,5-dithiourea

We have developed for the first time a robust acid-resistant chelating polymer (25DTF) for enhanced stabilization of Pb^2+^ in fly ash (Scheme 1). 25DTF was synthesized by reacting formaldehyde with 2,5-dithiourea. The monomer contains two carbon–sulfur bonds, resulting in a significantly higher chelating capacity for Pb^2+^. The chelation efficiency of 25DTF for Pb^2+^ in fly ash was exceptionally high, reaching 100%. Importantly, there was no risk of secondary dissolution of Pb^2+^, even under acidic conditions. 25DTF has exhibited excellent chelation efficiency for Pb^2+^ across different regions, making it highly promising for applications in soil and wastewater treatment.

## Material and methods

### Materials

2,5-Dithiodiurea (AR, CAS:142-46-1) was procured from the Aladdin Reagent Company, formaldehyde (AR, CAS: 50-00-0) from the Xi’an Chemical Reagent Company, sodium hydroxide (AR, CAS:1310-73-2) was procured from the Damao Chemical Reagent Company and hydrochloric acid (AR; CAS:7647-01-0) from the Chengdu Kelon Reagent Company. MSWI fly ash from Hebei, Shandong, Guangdong and Sichuan were provided by Chengdu Heng Xinhe Environmental Protection Technology Company.

### Synthesis of 25DTF

**Fig. 1:**
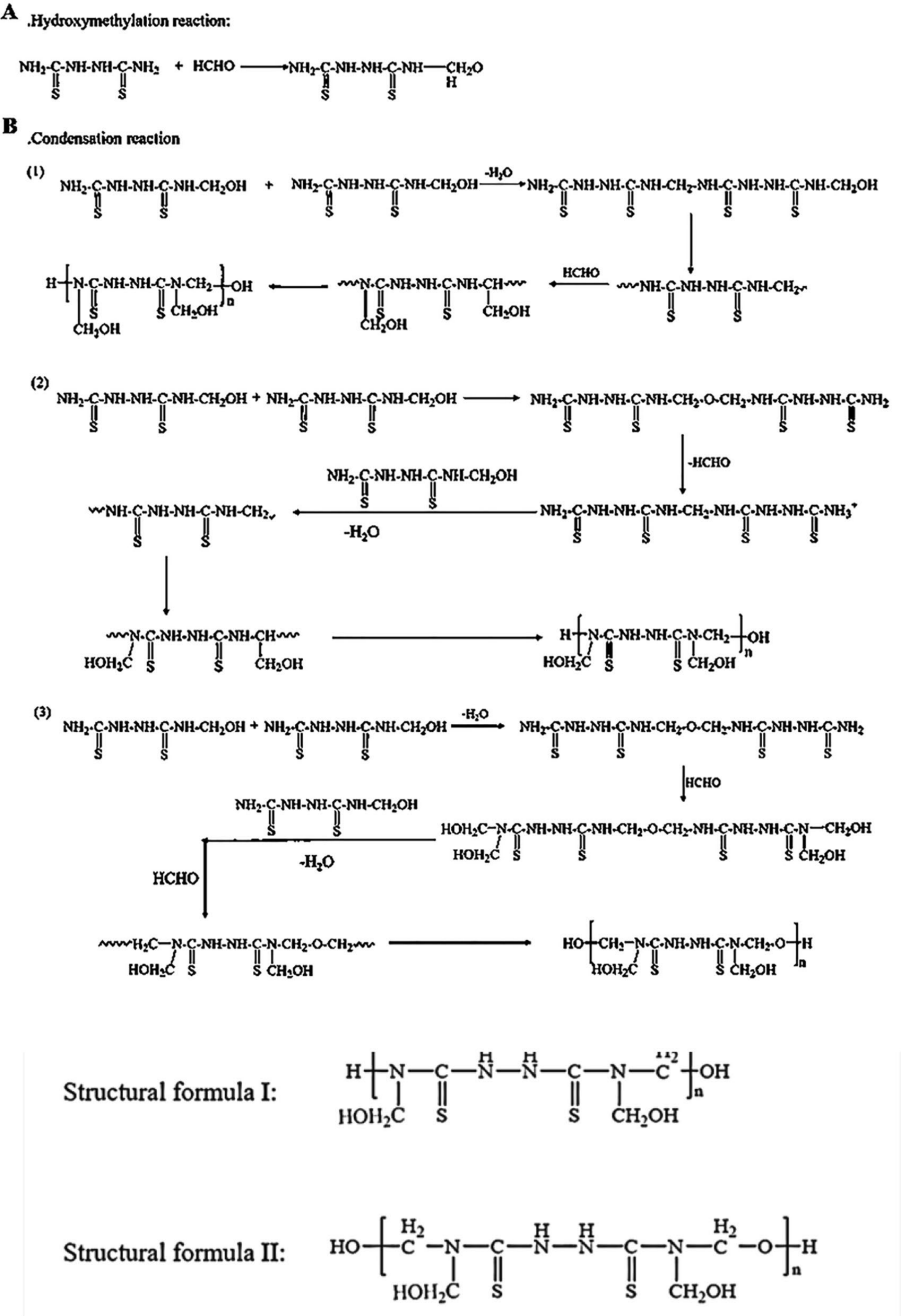
Synthetic route of 25DTF. The synthesis of the 25DTF resin involved amine formaldehyde reactions, which included hydroxymethylation (**A**) and condensation (**B**) processes

The synthesis procedure for 25DTF was conducted (Fig. 1): A three-necked round-bottom flask was charged with a mixture of 7.5 g of 2,5-dithiodiurea and 4.6 mL of formaldehyde. The mixture was heated with magnetic stirring (Speed Control 85–2 type constant temperature magnetic stirrer manufactured, Shanghai Silo Instrument Company), and the pH was adjusted to 9 by adding a 20% NaOH solution. Subsequently, the mixture was stirred for 30 min at 60 °C. Then, the pH was lowered to 5 by adding a 20% HCl solution, and the reaction mixture was stirred in an oil bath at 80 °C for 5 h. Once the reaction was complete, the mixture was cooled to room temperature and then dried to obtain 25DTF.

The 25DTF resin was synthesized through the amine-formaldehyde reaction, which included processes of hydroxymethylation (A) and condensation (B). During the condensation process, hydroxymethyl compounds led to the formation of either methylene or dimethylene ether bridges. Consequently, 25DTF existed in two structural forms.

### Characterization of 25DTF

Nuclear magnetic resonance spectroscopy (NMR) analysis was conducted to verify the successful synthesis of 25DTF. NMR images of 25DTF were recorded by using a JNM-ECS 400 M (JEOL, Japan), 25DTF (50 mg) was dissolved of in 0.5 mL of DMSO-d_6_. (^1^H RF frequency: 399.782MHZ; ^13^C RF frequency: 100.525MHZ. Contact time: 2.0 ms, Number of scans: 1024, Delay time: 5 s, Sampling time: 34.5 ms)).

Fourier-transform infrared (FT-IR) spectra were conducted to confirm the successful preparation of 25DTF using a Nicolet NEXUS 670 infrared spectrophotometer (Nicolet, NEXUS 670, USA). 1 mg of 25DTF was weighed and then mixed with 100 mg of dry KBr, and subsequently compacted into a 10 mm transparent sheet using a tablet press. FT-IR spectra were obtained from the sheet in the range of 400 to 4000 cm^−1^ with a data acquisition rate of 4 cm^−1^ per point and Automatic smoothing.

Raman spectrometer was employed to assess the successful preparation of 25DTF. Specifically, 20 mg of 25DTF was directly compressed into a tablet from the powder sample, and then placed on a slide for analysis using the Lab RAM HR Evolution instrument (HORIBA FRANCE SAS, France) to characterize the 25DTF.

### The removal effect of 25DTF on Pb^2+^

35 g of fly ash was dissolved in 100 mL of ultrapure water. After centrifugation and filtration of the supernatant, a measured volume of the resulting liquid was taken, and 1 mL of a 25DTF solution (100 mg/mL) was added. The pH was adjusted to 7. After centrifugation, the supernatant was passed through a 0.22 μm filter membrane, and the content of heavy metal ions was determined by an Inductively Coupled Plasma-Optical Emission Spectrometer Inductively Coupled Plasma-Optical Emission Spectrometer (ICP-OES) (Plasma Quant PQ9000, Germany). The data are presented as the mean ± standard deviation (SD) of three experiments.

### The secondary leaching of Pb^2+^

The fly ash filtrate was mixed with a solution containing 25DTF at a concentration of 100 mg/mL and adjusted to pH 7 before agitation and subsequent centrifugation at 10,000 rpm/min. The concentration of Pb^2+^ in the resulting supernatant was measured. The precipitate obtained was then mixed with 5 mL of water, and the pH was adjusted to 4. After another round of centrifugation at 10,000 rpm/min, the Pb^2+^ concentration in the filtrate was measured again. The data are presented as the mean ± standard deviation (SD) of three experiments.

## Results and discussion

### Characterization of 25DTF

**Fig. 2:**
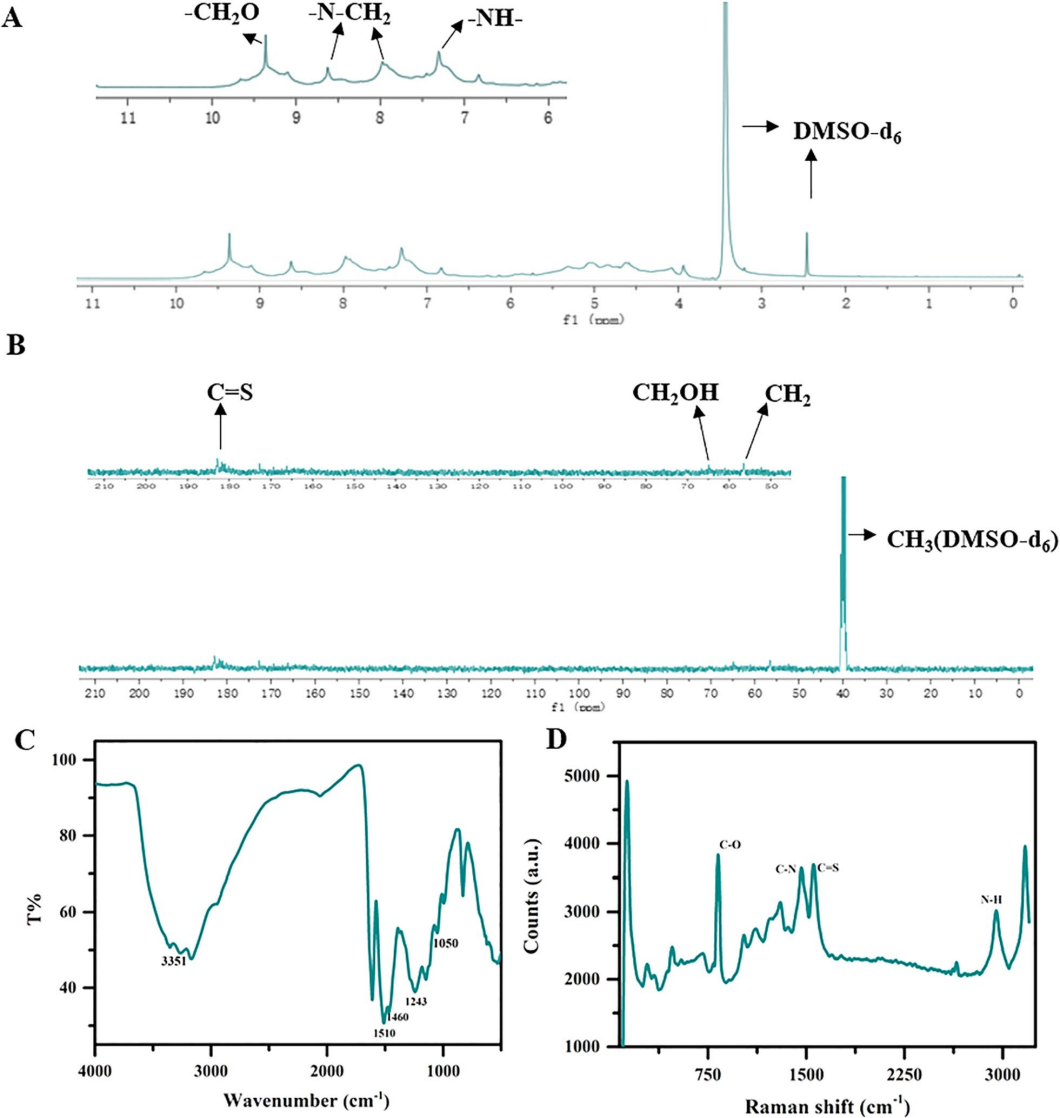
25DTF NMR spectrum: the ^1^H NMR of the 25DTF (**A**); the ^13^C NMR of the 25DTF (**B**); FTIR (**C**) and Raman (**D**) spectra of 25DTF

The synthesis of 25DTF was evaluated by NMR spectroscopy (Fig. 2A, B). In the ^1^H NMR spectrum, the peak at 2.5 ppm was attributed to DMSO-d_6_, while the peak at 9.8 ppm corresponded to the -CH_2_-O bond. Additionally, the characteristic peak of N-CH_2_- was observed at 8.66 ppm. Furthermore, peaks at 7.3 ppm and 8.0 ppm represented the characteristic peaks of -NH- and -OH, respectively (Fig. 2A). The ^13^C NMR depicted a prominent C = S characteristic peak at 182 ppm, a characteristic peak of -CH_2_OH at 65 ppm, and another characteristic peak of -CH_2_- at 56 ppm. The peak at 40 ppm was attributed to DMSO-d_6_ (Fig. 2B). Additionally, the analysis revealed two forms of methylene groups in the prepared 25DTF: -CH_2_OH and -CH_2_-. Both exhibited negative signal peaks in the DEPT 135 spectrum (Figure S1). These findings suggested that the prepared 25DTF existed in two structural forms.

The synthesized 25DTF was characterized by FTIR and Raman spectroscopy (Fig. 2C, D). In the FTIR analysis, notable peaks for 25DTF were observed (Fig. 2C). The characteristic peaks of 25DTF were assigned at 3351 cm^−1^ for N–H and H–O stretching, and 1050 cm^−1^ for C–O–C stretching. The peak recorded was at 1510 cm^−1^ for N–C = S stretching. In addition, the peaks at 1460 cm^−1^ correspond to N–C stretching. The peaks at 1243 cm^−1^ in 25DTF were for C-H stretching of N–CH-N < .

The Raman spectrum of 25DTF exhibited a strong peak associated with the vibration of the C-O bond at 825 cm^−1^, while the N–C bond was clearly represented by peaks at 1467 cm^−1^. Notably, a distinctive absorption peak near 1554 cm^−1^, attributed to the C = S bond, was observed. Additionally, the peak at 3171 cm^−1^ corresponded to the N–H bond (Fig. 2D). These results confirmed the successful synthesis of 25DTF.

### The effect of concentration of 25DTF on the chelation of Pb^2+^

**Fig. 3:**
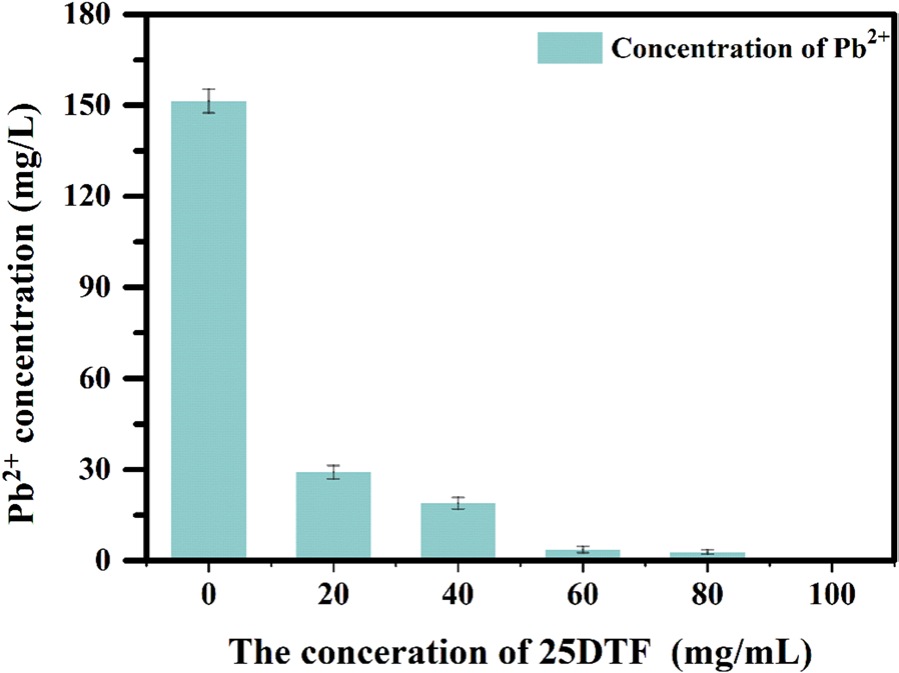
The effect of concentration (0, 20, 40, 60, 80, and 100 mg/mL) of 25DTF on the chelation of Pb^2+^

The impact of 25DTF concentration on the chelation process was assessed through ICP-OES analysis (Fig. 3). It was observed that as the concentration of 25DTF increased, the concentration of Pb^2+^ in the fly ash decreased progressively. When the 25DTF concentration reached 100 mg/mL, the residual Pb^2+^ concentration in the treated fly ash was (3 × 10^–4^ ± 0.05) mg/L, significantly lower than the landfill standard of 0.25 mg/L, rendering it suitable for disposal. This result underscored the robust chelating capability of 25DTF for Pb^2+^. This could be attributed to the presence of covalently bound functional groups in the chelating agent, which contain nitrogen and sulfur atoms capable of forming stable complexes with metal ions.

### The chelation capacity of 25DTF at pH ≤ 7

**Fig. 4:**
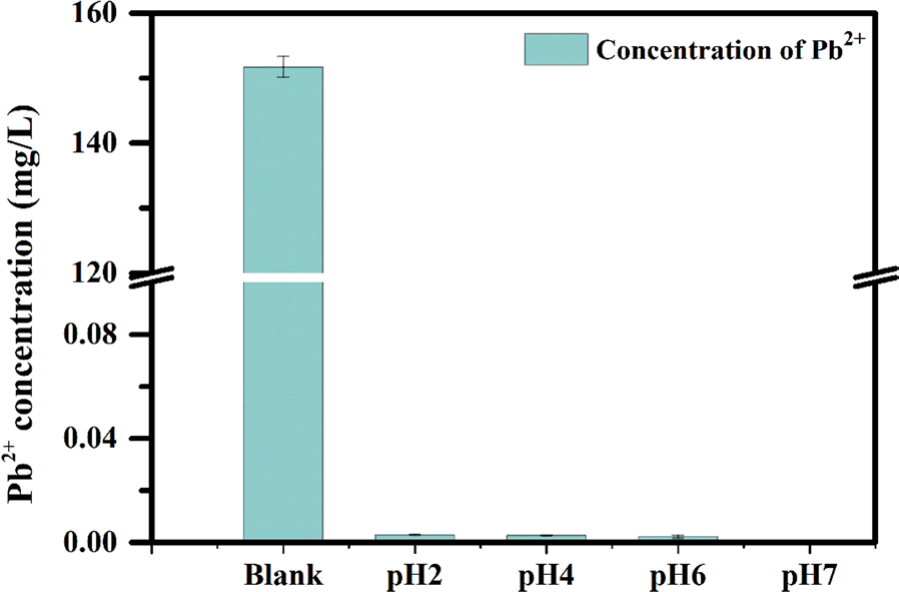
The chelating effect of 25DTF on Pb^2+^ under pHs (2, 4, 6, 7)

The chelation capacity of 25DTF at pH ≤ 7 was investigated through an ICP-OES experiment (Fig. 4). The initial concentration of Pb^2+^ in untreated fly ash was (151.7 ± 1.6) mg/L. Upon the addition of 25DTF, the residual Pb^2+^ concentrations in the treated fly ash at pH levels 2, 4, 6, and 7 were measured as (2.9 × 10^–3^ ± 9 × 10^–6^) mg/L, (2.7 × 10^–3^ ± 7 × 10^–5^) mg/L, (2.2 × 10^–3^ ± 5 × 10^–4^) mg/L, and (3 × 10^–4^ ± 4 × 10^–4^) mg/L, respectively. This observation clearly demonstrated that 25DTF achieved an outstanding chelation efficiency of over 99.9% when interacting with Pb^2+^ in fly ash under pH ≤ 7.

### The ability to chelate Pb^2+^ of different chelating agents

**Fig. 5:**
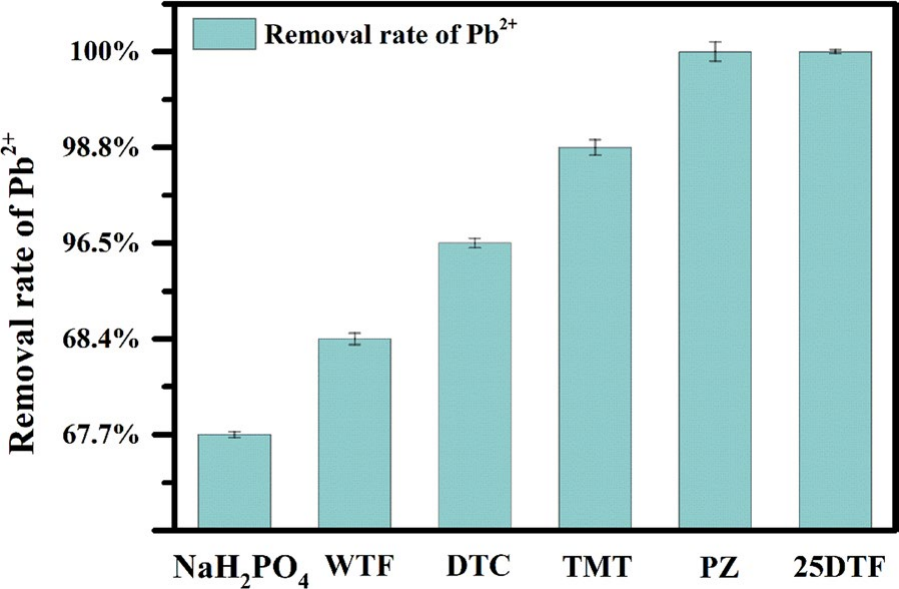
Removal rate of Pb^2+^ in fly ash with the different chelating agent (NaH_2_PO_4_, WTF, DTC, TMT, PZ, and 25DTF)

To investigate the chelation capability of different chelating agents for Pb^2+^ in fly ash, an ICP-OES experiment was conducted (Fig. 5). Subsequently, 100 mg each of NaH_2_PO_4_, Di-thiocarbamate (DTC), 1,3,5-Triazine-2,4,6-trithiol trisodium salt (TMT), WTF, and 25DTF were added to 5 mL of fly ash filtrate. The chelation rates were recorded as follows: NaH_2_PO_4_ at (66.7 ± 3)%, DTC at (96.5 ± 6)%, TMT at (98.8 ± 5)%, WTF at (67.8 ± 8)%, PZ at (100 ± 10)% and 25DTF at (100 ± 2)%. This result demonstrated that 25DTF outperformed other chelating agents in efficiently capturing Pb^2+^ in fly ash. Organic sulfur and nitrogen compounds are commonly categorized as either soft or intermediate bases, whereas the majority of heavy metal ions are classified as intermediate acids, thereby facilitating the formation of stable chelates. Therefore, the presence of thioamide (-CS-N-) groups in 25DTF likely contributes to its excellent chelating effect. These groups enable 25DTF to bind with a larger quantity of Pb^2+^ in the mesh structure, effectively chelating Pb^2+^ in fly ash.

### The secondary leaching of Pb^2+^ chelated from different chelators

**Fig. 6:**
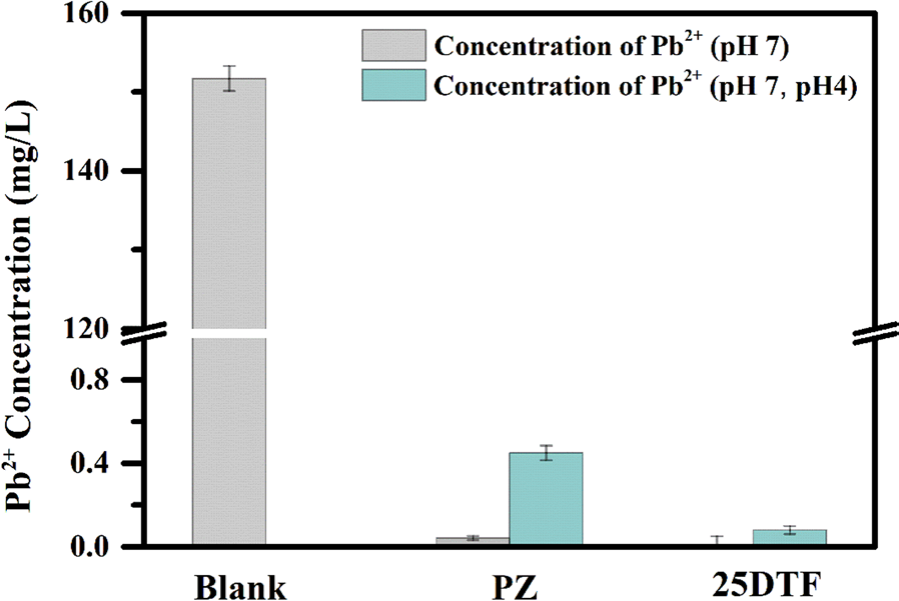
The secondary leaching of Pb^2+^ chelated by from different chelators (PZ and 25DTF). The concentration of Pb^2+^ at pH 7(grey), The concentration of Pb^2+^ at pH 7 and pH 4 (green)

The secondary leaching of Pb^2+^ chelated by different chelating agents was evaluated using ICP-OES (Fig. 6). The initial concentration of Pb^2+^ in fly ash was (151.7 ± 1.6) mg/L. After chelation by 25DTF at pH 7, the concentration of Pb^2+^ decreased to only (3 × 10^–4^ ± 0.05) mg/L. In contrast, the remaining Pb^2+^ concentration in the fly ash treated with Potassium piperazine-N, N'-bis(dithiocarboxylate) (PZ) was (0.04 ± 0.01) mg/L. Under acidic conditions (pH 4), the residual Pb^2+^ concentration in the fly ash treated with PZ increased to (0.45 ± 0.04) mg/L, whereas in the fly ash treated with 25DTF, it was significantly lower at (0.08 ± 0.02) mg/L. This finding highlighted the remarkable stability of 25DTF in chelating Pb^2+^, effectively preventing their secondary dissolution, particularly under acidic conditions.

### Application of 25DTF in practical samples

**Fig. 7:**
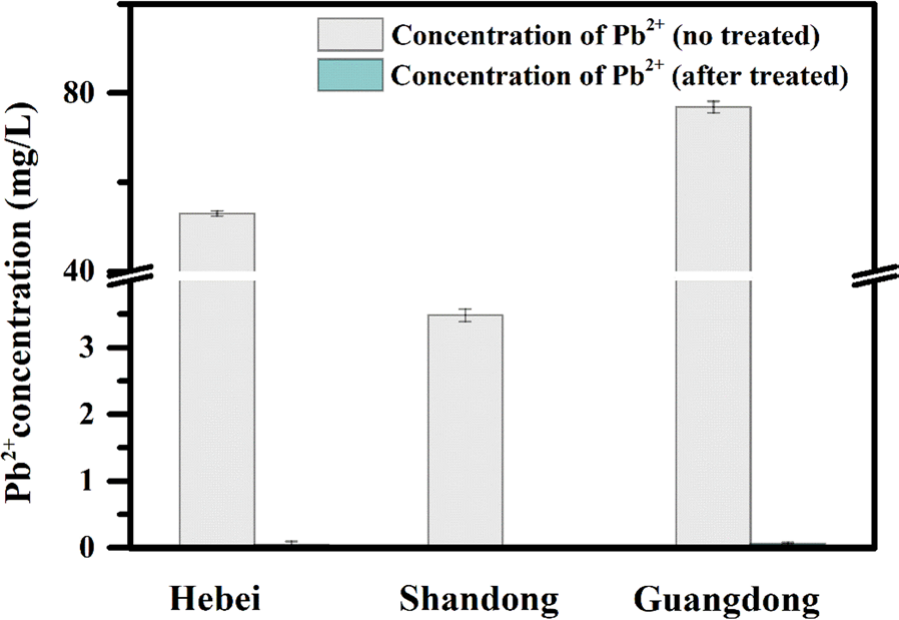
The chelation effect of Pb^2+^ in fly ash (no treated (grey) and after treated (green)) from different regions of China (Hebei, Shandong, Guangdong)

A comprehensive evaluation of the chelating effect of 25DTF on Pb^2+^ in fly ash from Hebei, Shandong, and Guangdong provinces was conducted using ICP-OES (Fig. 7). Following the chelation process, the Pb^2+^ concentration in the Hebei sample treated with 25DTF decreased significantly from (53.0 ± 6) mg/L to a minimal (0.04 ± 0.05) mg/L. Similarly, in the Shandong sample, it dropped from (3.5 ± 0.1) mg/L to (0 ± 0.02) mg/L when treated with 25DTF. Likewise, in the Guangdong sample, the Pb^2+^ concentration decreased dramatically from (76.8 ± 1.3) mg/L to a mere (0.07 ± 0.01) mg/L when treated with 25DTF. This result suggested the potent chelating effect of 25DTF on Pb^2+^ presented in fly ash samples from diverse regions in China.

## Conclusion

Municipal solid waste incinerator (MSWI) technology has garnered significant attention in waste treatment due to its superior treatment capacity, excellent waste reduction capability, and comprehensive environmental safety measures. However, the fly ash generated by MSWI contains numerous heavy metals with high leaching toxicity, posing significant environmental concerns. Pb^2+^ is a globally recognized toxic pollutant that poses risks to both the environment and human health. Cement solidification and melt solidification methods for the treatment of Pb^2+^ in fly ash suffer from drawbacks such as increased volume and high cost. The chemical stabilization method using chelating agents is cost-effective and widely accessible. However, its efficacy in stabilizing Pb^2+^ under acidic conditions is limited. Therefore, there is a critical need to develop a novel chelating agent capable of efficiently removing Pb^2+^, particularly under acidic conditions.

The novel polymeric chelating agent 25DTF was synthesized by reacting 2,5-dithiourea and formaldehyde. Its successful preparation was confirmed through analysis using NMR, FTIR, and Raman spectra. Furthermore, the chelation between 25DTF and Pb^2+^ displayed remarkable stability and remained unaffected by acidic conditions, achieving a chelation rate close to 100% across a wide pH range. Additionally, 25DTF exhibited superior chelation of Pb^2+^ in fly ash samples sourced from various regions, highlighting its substantial potential for environmental remediation purposes. The 25DTF was predominantly comprised of alkali organic compounds containing sulfur, with Pb^2+^ serving as intermediate acids. The principle of acid–base complementarity was utilized to link the chelating agent with the coordination bond of Pb^2+^, thereby facilitating the formation of a robust grid structure, restraining the leaching of Pb^2+^, and decreasing the Pb^2+^ content in fly ash. The novel chelating agent holds promising application prospects for remediating metal ions in soil and wastewater, thereby contributing to the advancement of municipal waste treatment practices.

In this study, progress has been made in the treatment technology of fly ash from domestic waste incineration. It was worth noting that the different of Pb^2+^ content in fly ash samples could be attributed to the composition of household waste generated in the respective regions. The synthesis process of the new polymer chelator 25DTF in our study involved the introduction of formaldehyde, the residue of which may induce toxicity. Additionally, the synthesis process required regulation of pH, and the reaction conditions were harsh. Hence, there is a pressing need to devise simple, environmentally friendly, safe, and stable chelating agents. In future studies, addressing the removal of formaldehyde residues during the synthesis of 25DTF may become a focal point of our research. Exploring efficient and straightforward methods for eliminating formaldehyde residues will be crucial in this regard. Furthermore, there is potential to optimize the preparation process of 25DTF and explore the development of a simpler and more efficient method.

### Supplementary Information

Supplementary Material 1.

## Data Availability

The datasets used or analyzed during the current study are available from the corresponding author on reasonable request.
