# Supplementary material for: A robust acid-resistant chelating polymer for enhanced stabilization of lead ions in fly ash
Source: BMC Chem. 2024 May 23;18(1):103. doi: 10.1186/s13065-024-01209-z (PMC11119302; doi:10.1186/s13065-024-01209-z)
Supplement: Supplementary file 1 — Supplementary Material 1. [file 13065_2024_1209_MOESM1_ESM.docx]

**A robust acid-resistant chelating polymer for enhanced stabilization of lead ions in fly ash**

**Qi Wang^1^, Huiyu Yan^1^, Linyan Yao^1^, Ying Guo^1^, Jianxi Xiao^1*^**

**^1^ State Key Laboratory of Applied Organic Chemistry, College of Chemistry and Chemical Engineering, Lanzhou University, Lanzhou, Gansu, 730000**


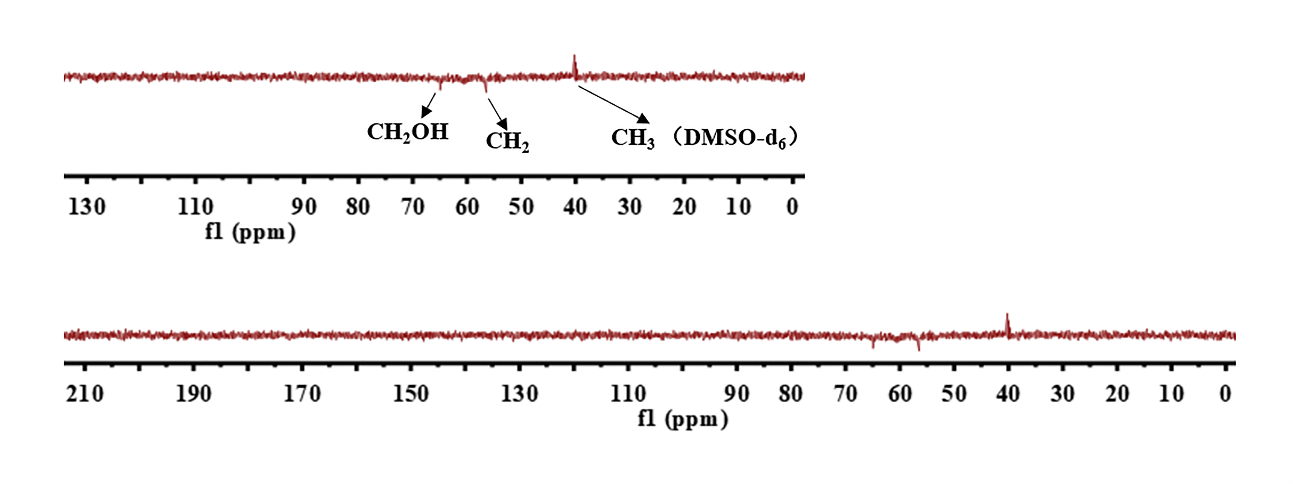
^*^Corresponding author: E-mail: xiaojx@lzu.edu.cn (J. Xiao)

Figure S1 The DEPT135 NMR of the 25DTF.
